# Toward Transplantation of Liver Organoids: From Biology and Ethics to Cost-effective Therapy

**DOI:** 10.1097/TP.0000000000004520

**Published:** 2023-07-20

**Authors:** Marjolein J.M. ten Dam, Geert W.J. Frederix, Renske M.T. ten Ham, Luc J.W. van der Laan, Kerstin Schneeberger

**Affiliations:** 1 Department Clinical Sciences, Faculty of Veterinary Medicine, Utrecht University, Utrecht, The Netherlands.; 2 Department of Public Health, Healthcare Innovation and Evaluation and Medical Humanities, Julius Center, Utrecht University, Utrecht, The Netherlands.; 3 Department of Surgery, Erasmus MC-University Medical Center, Rotterdam, The Netherlands.

## Abstract

Liver disease is a common cause of morbidity and mortality, and many patients would benefit from liver transplantation. However, because of a shortage of suitable donor livers, even of those patients who are placed on the donor liver waiting list, many do not survive the waiting time for transplantation. Therefore, alternative treatments for end-stage liver disease need to be explored. Recent advances in organoid technology might serve as a solution to overcome the donor liver shortage in the future. In this overview, we highlight the potential of organoid technology for cell therapy and tissue engineering approaches. Both organoid-based approaches could be used as treatment for end-stage liver disease patients. Additionally, organoid-based cell therapy can also be used to repair liver grafts ex vivo to increase the supply of transplantable liver tissue. The potential of both approaches to become clinically available is carefully assessed, including their clinical, ethical, and economic implications. We provide insight into what aspects should be considered further to allow alternatives to donor liver transplantation to be successfully clinically implemented.

## INTRODUCTION

Every day, around 17 people in the United States and 21 people in Europe die while waiting for their life-saving donor organ.^1,2^ In fact, the number of people on the donor organ waiting list steadily increases. Every 10 min, someone is added to the national waiting lists, both in the United States and Europe.^2,3^ Of all organs, livers are the second most required organs for transplantation purposes.^4^ It is estimated that 1.5 billion people are having chronic liver disease worldwide, including viral hepatitis (hepatitis B virus and hepatitis C virus), hepatocellular carcinoma (HC), nonalcoholic fatty liver disease, nonalcoholic steatohepatitis, and alcohol-associated liver disease.^5,6^ Many of these diseases lead to liver fibrosis, which can rapidly result in cirrhosis and end-stage liver disease (ESLD).^7,8^ Over the years, the global burden of HC and cirrhosis has rapidly increased, making ESLD the 12th leading cause of death globally.^7,8^ Currently, the only curative treatment for ESLD patients is liver transplantation. However, the rising demand far exceeds the number of available donor livers, resulting in a large discrepancy between the number of donors and recipients.^9^ This discrepancy is both because of the rapidly rising demand for, and the decreasing availability of, suitable donor livers. Medical progress and superior living conditions have led to an increase in people reaching advanced age, which leads to a significant increase in the donor pool’s age as well. However, with advanced age there is also a higher prevalence of age- and lifestyle-related diseases, which remarkably increases the need for new donor livers and also hampers the transplantable use of these livers postmortem.^10^ It is expected that the number of people in need of liver transplantation is likely to increase further because of long-term harmful effects of specifically hepatitis B virus and increasing prevalence of people having obesity with its adverse effects on liver function.^11^ Because of this gap in supply and demand, the selection criteria for a patient to enter the donor organ waiting list are very stringent. The validated scoring system called the Model for End-stage Liver Disease (MELD) score is one way to determine whether patients are eligible for transplantation. The MELD score rates the urgency for a new liver by estimating the chances of disease survival for the next 3 mo.^12^ Yet, even if patients are classified with a high-priority MELD score, many must still wait for 1 or 2 y before receiving their life-saving donor liver. Because of this waiting time, many patients are unable to survive the donor liver waiting list.^11,13^ Even worse, waiting list mortality is presumably an underestimation of the donor liver shortage because most people are not even listed and die without being offered a liver transplant.

**FIGURE 1.**
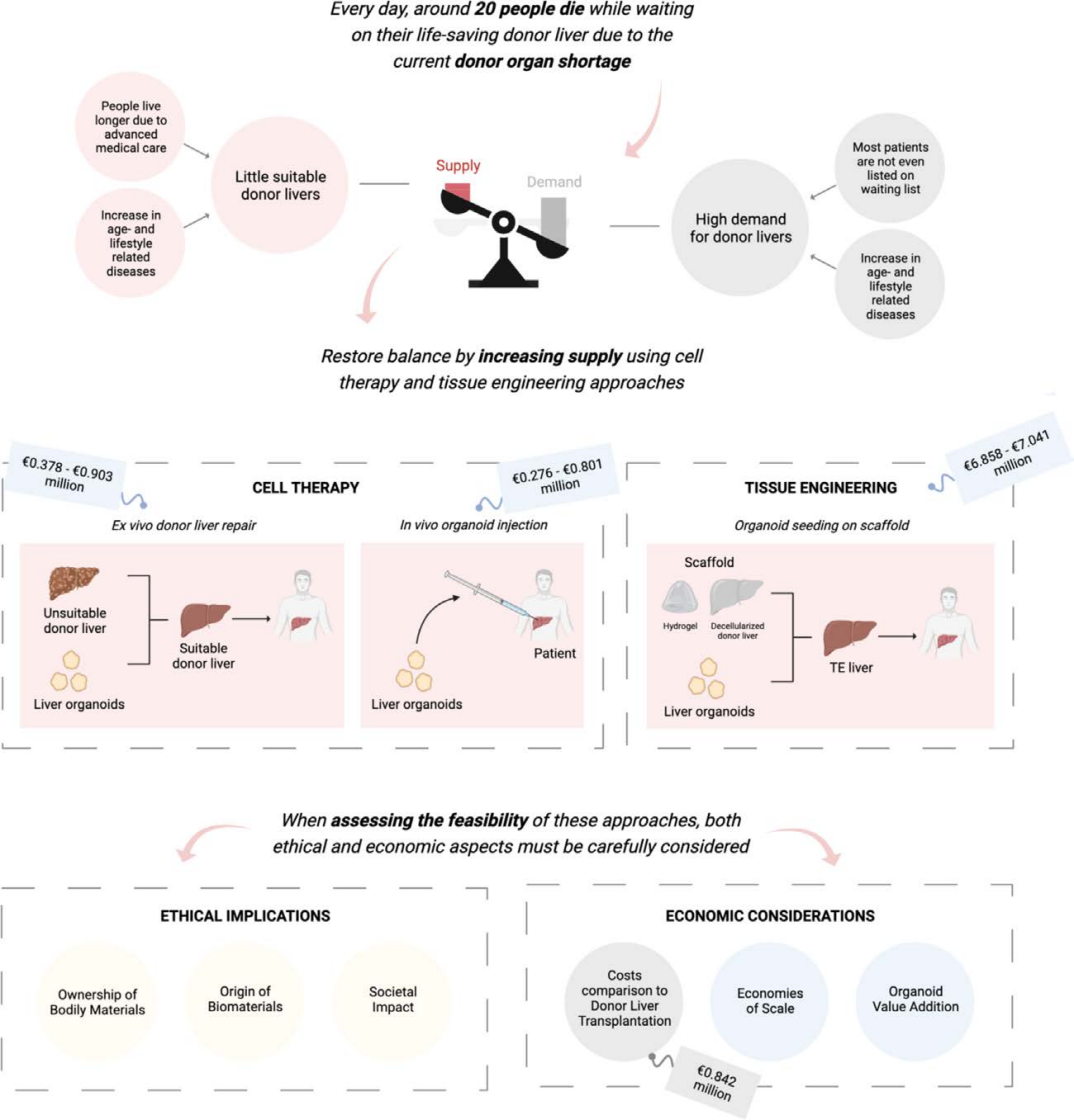
Graphical abstract on the use of cell therapy and tissue engineering approaches to tackle the donor organ shortage problem, including ethical and economic implications that must be considered when assessing clinical feasibility. Created with BioRender.com.

The problem of donor liver shortage might be tackled by liver regenerative therapy (LRT), in which methods are developed to replace diseased liver tissue. Many patients, including those who do not qualify for the waiting list, would greatly benefit from alternative treatment options, including cell therapy (CT) and epigenetic or gene therapies for patients having genetic disorders. Currently, CT is performed with primary human hepatocytes (PHHs) to restore failing liver function and alleviate disease symptoms. However, high-quality PHHs are scarce, as they are isolated from donor livers deemed unsuitable for transplantation because of excessive steatosis, prolonged ischemia time, high donor age, or cardiac arrest.^14^ Therefore, organoids have been explored as an alternative cell source for CT and tissue engineering (TE) that aim to overcome the donor liver shortage by either creating new liver tissue or by repairing donor livers unsuitable for transplantation ex vivo.^15,16^ This overview highlights the opportunities and limitations of organoid technology for LRT, by first introducing the advances made in organoid technology, followed by their use for CT and TE. Subsequently, we will evaluate which ethical and economic challenges must be addressed before the clinical application of organoids is feasible. Figure 1 provides a graphical overview on the supply and demand gap that could potentially be decreased with the use of organoid-based CT and TE approaches.

## LIVER ORGANOIDS AND THEIR THERAPEUTIC APPLICATIONS

**FIGURE 2.**
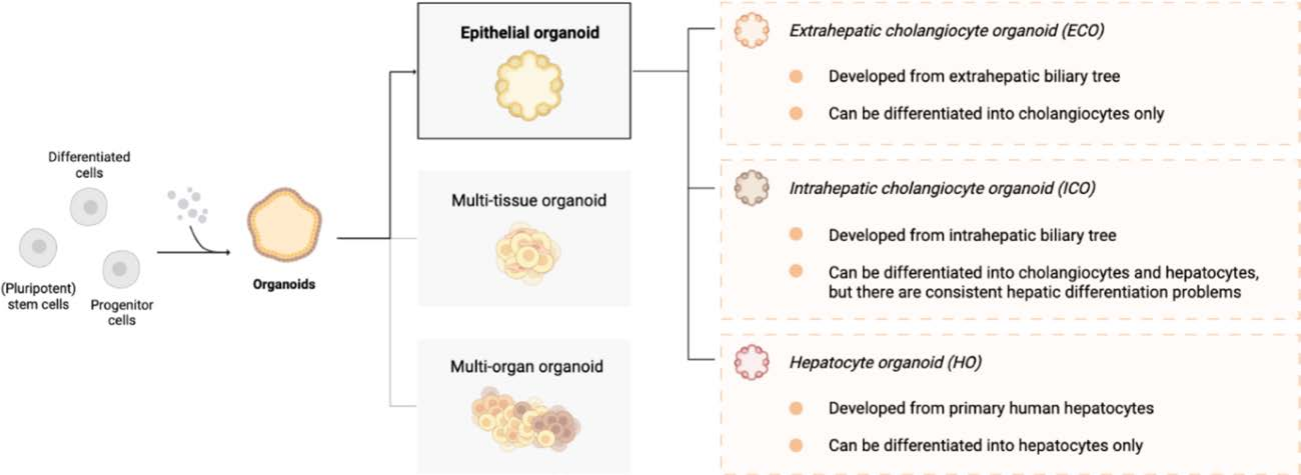
Graphical overview depicting the nomenclature of different liver organoids. Organoids can be derived from differentiated cells, (pluripotent) stem cells, or progenitor cells. The 3 main types of organoids are epithelial, multitissue, and multiorgan. Focused on epithelial organoids, 3 main types of liver organoids are known: ECOs, ICOs, and HOs, referring to their cell type of origin. Figure is adapted from Marsee et al.^14^ Created with BioRender.com. ECO, extrahepatic cholangiocyte organoid; ICO, intrahepatic cholangiocyte organoid; HO, hepatocyte organoid.

In recent years, major breakthroughs have been achieved in expanding organoids. As recently described by Marsee et al^17^ an organoid is defined as: “A three-dimensional structure derived from stem cells, progenitor, and/or differentiated cells that self-organizes through cell–cell and cell–matrix interactions to recapitulate aspects of the native tissue architecture and function in vitro.” Figure 2 provides a graphical overview on the nomenclature of the different liver organoids. In this overview, we will focus on epithelial organoids derived from parenchymal cells isolated from the adult liver, as it is a widely studied organoid type able to self-renew.^18^ Both primary cholangiocytes and hepatocytes have been used as a cellular source to establish organoids.^18-21^ Cholangiocytes constitute 3% to 5% of the total liver mass and represent a heterogenous, dynamic population of epithelial cells that line the biliary tree and are crucial to bile secretion out of the liver.^22^ In 2013, a landmark article by Huch et al^18^ first demonstrated that Leucine-rich repeat-containing G-protein coupled Receptor 5+ progenitor cells from murine origin can create organoids in vitro. Two years later, Huch et al^19^ demonstrated organoid development from biliary progenitor cells from human liver tissue. Organoids derived from cholangiocytes of the adult intrahepatic biliary tree are termed intrahepatic cholangiocyte organoids (ICOs), which are bipotential, and after expansion can be differentiated into either cholangiocyte-like or hepatocyte-like cells. Organoids can also be established from the extrahepatic bile ducts and gallbladder, which are termed extrahepatic cholangiocyte organoids (ECOs) and gallbladder cholangiocyte organoids, respectively. Recent single-cell atlas disclosed extensive heterogeneity of liver stem cell niches and demonstrated that ICOs, ECOs, and gallbladder cholangiocyte organoids are notably similar, but show some regional differences related to their native anatomical location.^23,24^ Unlike ICOs, ECOs lack a bipotential fate and can only be efficiently differentiated into cholangiocytes, not hepatocytes.^24,25^ However, even for ICOs, current differentiation protocols are insufficient in differentiating ICOs toward a mature hepatocyte phenotype, which consequentially leads to impaired hepatic function. Hepatocytes account for 70% of the total liver cell mass and are key to vital processes, such as detoxification, metabolization of glucose- and lipids, and albumin production.^20^ During the past 4 y, efforts have been made not only to improve hepatic maturation of ICOs, but also to establish organoids directly from hepatocytes, termed hepatocyte organoids (HOs).^20,21,25,26^ In 2018, Hu et al^20^ developed long-term culture conditions for HOs from mature murine hepatocytes, human fetal liver cells, and cryopreserved PHHs. HOs express markers related to liver regeneration typically observed after partial hepatectomy, which suggests that in vitro expansion might be mimicking in vivo regenerative processes.^20,24^ Yet, excitement must be mitigated, because until now, only long-term culture conditions for functional HOs of murine or human fetal origin have been sufficiently established.

For LRT, organoids can be used on their own or in combination with scaffolds such as natural or synthetic biomaterials and decellularized livers.^20,27-30^ Scaffold-encapsulated cells allow for an adequate representation of the native liver structure. Here, technical opportunities and challenges of both CT (focused on cell transplantation) and TE (focused on transplantation of tissue made of cells combined with scaffolds) will be discussed.

### Organoids for CT

We expect that patients having diseases related to a metabolic defect of a single-cell type can be treated best with organoid-based CT, restoring solely the failing part of the liver. For instance, in patients having cholangiopathies, like primary biliary cirrhosis and primary sclerosing cholangitis, sclerosis narrows the bile ducts causing harmful bile buildup.^31,32^ We hypothesized that bile duct function can be restored by infusion of autologous or allogeneic ICO or ECO cells, depending on the location of the sclerosis.^33-35^ One way to deliver the organoids into the intrahepatic biliary tree is via common bile duct injection—a protocol developed by Berntsen et al in 2018, which has been successfully used by Sampaziotis et al as well.^35-37^ Potential challenges for this approach are that fibrosis/sclerosis limits organoid cell engraftment and that autoimmune responses might cause recurrence of pathogenesis. CT can also be used for ex vivo regeneration of rejected donor livers. Approximately 20% of donor livers are excluded from the donor pool because of bile duct damage and CT could be used to improve donor liver quality, allowing them to pass the quality-control check.^37^ Recently, both ICOs and ECOs have been used to repair the intrahepatic and extrahepatic bile ducts in discarded donor livers.^37^ Approximately 10 million organoids were infused into the common bile duct, and after 100 h of machine perfusion, 60% of the organoids were still present and total bile volume and pH were significantly increased compared with the control group.^37^ Furthermore, a crucial clinical requirement for using any cell source relates to its ability to preserve a robust genetic and epigenetic status.^19^ For many cells, including induced pluripotent stem cells, genetic instability, and epigenetic anomalies that occur during cellular reprogramming raise concerns regarding their oncogenic potential and, thus, clinical safety.^38,39^ Yet, the establishment of ICOs does not require genetic reprogramming and extensive analysis by Huch et al^19^ showed that ICOs maintain their genetic integrity for at least 3 mo in vitro.^19^ Additionally, cholangiocyte organoids can also be generated from extrahepatic bile collected from resected gallbladders. These bile-derived cholangiocyte organoids show similar features to in vivo cholangiocytes and can sufficiently repopulate decellularized extrahepatic biliary grafts.^28^ However, these repaired grafts have not been transplanted in humans, so successful engraftment has not been proven yet.^27,28^

For patients having inherited liver diseases, like Wilsons disease, Crigler-Najjar, and α1-antitrypsin deficiency, typically, the hepatocytes are the most affected cell type in the liver.^40-42^ Therefore, we hypothesize that transplanting ICOs or HOs would be sufficient for these patients. Organoids can be delivered to the liver parenchyma via portal vein injection,^27^ which is also the preferred method for PHH transplantation.^43^ In patients with Crigler-Najjar, hepatocytes are unable to properly convert and clear bilirubin from the blood—a normal byproduct of hemolysis. Therefore, the aim of transplanting organoids will not be to repopulate the entire liver, but small numbers of hepatocyte-like cells able to clear bilirubin from the blood might be able to alleviate disease symptoms. Because there are no clinical data available on organoid transplantation, it is unclear how many organoids will be sufficient. Yet, past PHH transplantation has shown that up to 50% of the blood bilirubin levels could be reduced when transplanting 7.5 billion PHHs. However, most of these patients underwent donor liver transplantation (DLT) within a year.^44-46^ For Wilsons disease, the organoids must be able to repopulate the liver parenchyma to such a degree that the hepatocyte injury is resolved and normal liver function is restored, which would require a proliferative advantage of the transplanted cells.^47^ A proliferative advantage might be intrinsic to the transplanted cells’ healthy phenotype, as seen in several rat studies, or can be induced by methods that precondition the recipient’s liver, such as partial hepatectomy, portal embolization, and liver irradiation.^48-52^ In all cases, the native hepatocytes are substantially damaged, to which transplanted cells respond by proliferating, thereby compensating for the loss of native hepatocytes.^50-52^ For a1-antitrypsin deficiency, an RNA interference therapy has recently been investigated in a clinical phase II trial that might replace the need for CT for those patients.^53^

In 2015, human ICOs were first transplanted into immunocompromised BALB/c nude mice. Human albumin and α1-antitrypsin levels were found in the mice’ serum within 7 to 14 d; however, the liver volume reconstitution was remarkably low.^19^ In 2020, repeated ICO transplantations were performed in *COMMD1*-deficient dogs via portal vein injection. The organoids were autologous and genetically repaired for the *COMMD1* deficit. Although engraftment was low, the transplanted cells survived for up to 2 y posttransplantation, which shows the potential of CT and also underlines the need for further optimization of organoid engraftment.^27^ HOs potentially have an engraftment advantage over ICOs in the liver parenchyma because HOs have a more mature hepatocyte phenotype that might facilitate efficient engraftment by improved cell–cell and cell–matrix interactions.^20^ A recent study has demonstrated the in vivo regenerative capacity of PHH-derived HOs posttransplantation in Fah^–/–^ mice.^21^ The engrafted HOs were able to repopulate the host liver for 80% 100 d posttransplantation. The HOs displayed the hepatocyte marker hepatocyte nuclear factor 4-α and did not express biliary markers keratin 19, SRY-box transcription factor 9, or keratin 7. The HOs also reestablished zonation marker expression, which was not detected in the host tissue pretransplantation. These findings suggest that the expression profile of transplanted HOs is determined by the host liver microenvironment.^21^ Another study, performed by Hu et al, demonstrated human fetal liver-derived HO transplantation in Fah^–/–^Rag2^–/–^Il-2ry^–/–^ mice.^20^ Although in vivo maturation was observed, limited engraftment of transplanted HOs was shown. Additionally, a comparison between fetal- and PHH-derived HOs showed that PHHs-derived HOs significantly outperformed fetal-derived HOs in terms of proliferation and engraftment. This suggests that PHH-derived HOs are better suited for CT.^20^

### Organoids for TE

**FIGURE 3.**
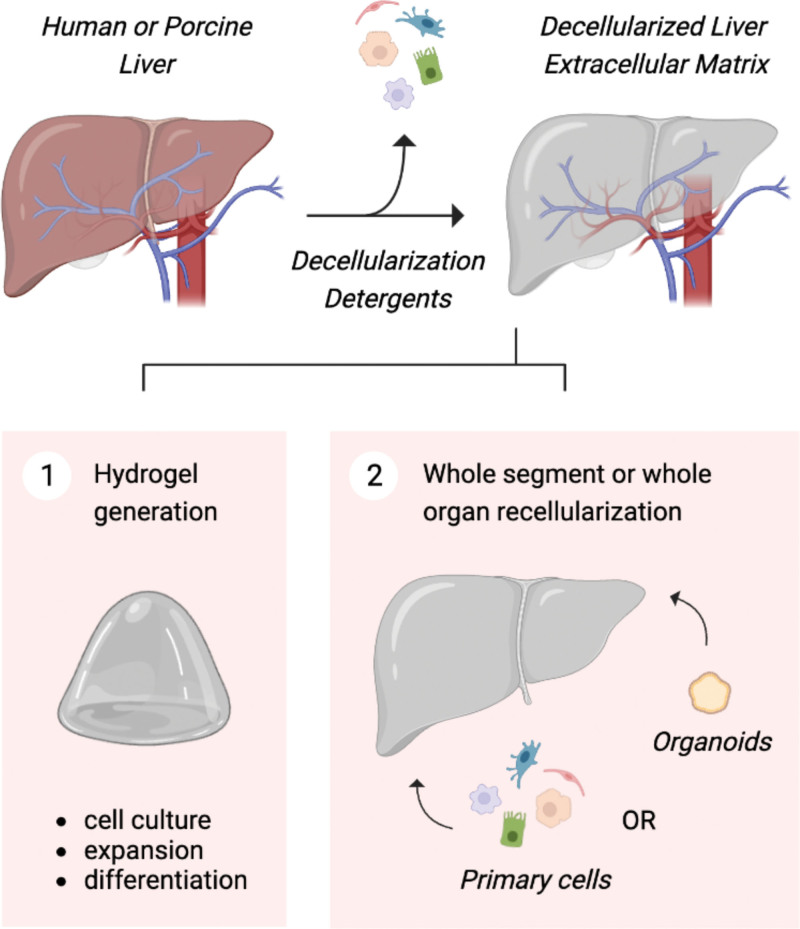
Decellularization of human livers using controlled perfusion via the hepatic artery and vena porta. Decellularized liver tissue can be used to generate a hydrogel for in vitro organoid cultures or for repopulation with ICOs, ECOs, HOs, or a combination thereof. Created with BioRender.com. ECO, extrahepatic cholangiocyte organoid; ICO, intrahepatic cholangiocyte organoid; HO, hepatocyte organoid.

TE aims to create functional tissues from cells combined with a scaffold consisting of either biological or synthetic biomaterials, mostly hydrogels or decellularized organs. A wide variety of biomaterials has been used, and for a concise overview of suitable hydrogels for LRT, we refer to a review of Ye et al^54^ and a review of Willemse et al.^55^ Organoid-based TE would be the preferred option to treat diseases affecting the entire liver, like steatohepatitis and cirrhosis. Either a complete liver can be bioengineered, or a construct large enough to restore the failing liver function. In the case of a split donor liver procedure, only 1 lobe of the donor liver is transplanted, which then regenerates with a rapid increase in liver volume up to near normal liver volume.^56,57^ Therefore, we assume that a similar size of a bioengineered liver is sufficient to restore the failing liver function because of posttransplantation increase in liver volume. In most patients with HC, but not all, the underlying chronic liver disease is compensated, and liver functions are preserved. However, surgical resection of the tumor is limited by impaired regeneration capacities, especially in patients with cirrhosis, with a risk of liver failure after resection if the volume of the remnant livers is too small. TE could push the limits of surgical resection by compensating postoperative liver function. In this case, the size of the bioengineered liver tissue will be similar to the resected tissue size. Tissue constructs can be generated using either 3-dimensional (3D) bioprinting or organ de/recellularization, both of which will be discussed here. To arrange cells and hydrogels in a prepatterned 3D structure, 3D bioprinting is often applied, which allows to print small hepatic-like tissues.^58,59^ For example, Lee et al^58^ printed a collagen-based cell-laden hydrogel into a framework of polycaprolactone, in which the coculture and external structure provided a suitable cell environment, thereby increasing the in vitro survival and functionality of hepatocytes. Furthermore, a recent report showed that transplanted human 3D bioprinted liver structures prolonged the survival of mice with liver failure.^60^ However, in this study, hepaRG organoids were used, which is an immortalized cell line established from a liver tumor associated with chronic hepatitis and can therefore not be used in a clinical setting.^61^ Apart from bioprinting, recellularization of decellularized donor organs has gained much attention over the past years. Decellularized organs create a suitable environment in terms of biochemical and physical cues and allow for the creation of organs on a physiological scale.^62^ During decellularization, ultrastructural extracellular matrix (ECM) components, native microvascular network, and biliary drainage are preserved (Figure 3). Furthermore, no polymorphic HLAs remain after decellularization, suggesting that no allogeneic immune reaction will occur posttransplantation.^62^ Human donor livers that were deemed unsuitable for transplantation, but have an intact ECM, can be used for decellularization purposes. Decellularized livers can be used to generate hydrogels that are suitable for culture, large-scale expansion, and differentiation of human ICOs or can be directly repopulated with liver cells.^62,63^ In 2015, the first protocol to successfully decellularize and repopulate human livers with multiple hepatic donor cell lines was set up.^62^ A recent study demonstrated the repopulation of decellularized liver discs using ICOs. The ICOs repopulated the discs within 1 wk while remaining viable and adapting a columnar polarized shape.^64^ Additionally, Tomofuji et al^33^ showed successful reconstruction of the intrahepatic biliary tree in a rat decellularized liver by recellularization with human ICOs, which remained viable for more than a week. This provides evidence for the feasibility of using TE liver constructs for transplantation purposes. Yet, aiming at optimization for decellularization and recellularization of both model organisms and human donor tissue is crucial to ensure long-term functionality.^65^

## CHALLENGES WHEN USING ORGANOIDS FOR LRT

Organoids insufficiently mimic the natural, complex organ physiology because of 2 main reasons: (1) The organoids discussed here are purely epithelial, whereas organs are complex architectures harboring multiple cell types from different germ layers. (2) The absence of vascularization. Adequate vascularization is necessary to maintain normal oxygen levels in tissues. The provision of nutrients and oxygen in in vitro cultures is facilitated via diffusion, with a diffusive limit of 100 to 200 μm.^66^ Because soft-tissue organoids are typically cystic and have a single-cell layer that is no greater than the diffusive limit, they can easily grow up to a width of 4 to 5 mm.^64^ However, when organoids are used as building blocks for thicker tissues, vascular structures have to be incorporated to prevent the presence of a necrotic core. Decellularized livers can be used to create large tissues with a competent vascular network because vascular structures are preserved and can be repopulated with endothelial cells.^62^ Furthermore, this natural architecture might help to maintain a functional metabolic liver zonation, which is the consequence of nutrient and oxygen gradients along the periportal to pericentral axis and is fundamental for proper execution of all metabolic liver functions.^67^

Besides the challenges related to building complex tertiary structures, the interaction between the host environment and the transplanted tissue should be addressed as well. For both CT and TE, autologous and allogeneic organoids can be used to create liver tissue. It is important to outweigh the benefits and concerns when deciding which approach is most suitable. The most important advantage of autologous organoids is the absence of immunogenic compounds, which decreases the probability of immune rejection posttransplantation.^68^ However, a serious disadvantage of organoids from an autologous nature is that they must be expanded in vitro first and are therefore not readily available, which significantly delays the treatment process. Furthermore, in the case of genetic diseases, the genetic defect must be corrected, which further delays the treatment process. Opposed to that, allogeneic organoids can be cultured to clinically relevant numbers beforehand, making them off-the-shelf available.^68^ Yet, allogeneic organoids will elicit an immune response caused by the presence of HLAs, if the immune system is not suppressed by immunosuppression.^69^ Lifelong use of immunosuppression decreases a patient’s quality of life (QoL), increases the risks of adverse events related to drug toxicity, and significantly increases treatment costs. The use of gene editing might circumvent the need for immunosuppression because allogeneic organoids can be genetically manipulated to either knock-out the donor HLA or match the donor tissue’s HLA to the recipient. A recent study showed the use of clustered regularly interspaced short palindromic repeats–mediated genome editing to develop off-the-shelf, immune-compatible human embryonic stem cells (ESCs) with the aim of generating large quantities of ESC lines that cover a broad spectrum of HLA types.^70^ However, excitement must be mitigated because further optimization is required before we are able to match all HLA types. Furthermore, issues regarding the safety of clustered regularly interspaced short palindromic repeats technology should be considered, such as the onset of off-target effects (undesired changes in nontarget genome locations), and the potential risks that come with it.^71^

## ETHICAL IMPLICATIONS OF ORGANOID TECHNOLOGIES

The clinical implementation of a new treatment option has ethical implications, which—next to clinical and economic considerations—require due attention. In this section, we will discuss ownership of materials, irreversibility of transplantation, and the societal impact of new treatments.

### Ownership of Bodily Materials

Little literature has paid attention to the relevance of “ownership of bodily materials” for the clinical use of both CT and TE. When we look at organ donation, donors typically donate their tissue, whereas third parties might gain proprietary rights and profits.^72^ In the case of organoid development, we must consider that some organoids could be considered immortal because they can be expanded in culture for a seemingly infinite time. Therefore, they can be stored in living biobanks for global distribution. Although this improves scientific and clinical use, it might also give rise to unequal distribution of benefits.

A well-known example of using cell biopsies for scientific purposes is the story of Henrietta Lacks. In the year of 1951, a cell biopsy was taken from her to assess the presence of cancerous tissue. The cells were found to expand rapidly and indefinitely in vitro—something no one at that time had seen before. The death of Mrs Lacks that same year was seen as an opportunity to create a cell line without the need for her consent and led to the development of the now-widespread HeLa cell line. Although the case of Henrietta Lacks has been extensively condemned, today the “consent or anonymize” approach is still widely applied. Yet, the approach has become insufficient when it comes to storage and clinical/commercial use of human tissue.^73^ In this approach, donor consent is not necessary, provided that personal data and materials are anonymized. However, with the recent advances made in genomic sequencing, donors are more identifiable than ever.^74^ This “consent or anonymize” approach also relates to CT and TE in terms of biobanking organoids for transplantation purposes, specifically. Therefore, Boers and Bredenoord^75^ conclude that consent should be aimed at the context of future use instead of the research content only and to overcome ethical injustices, a benefit-sharing concept to ensure equal distribution of benefits is proposed. Additionally, ethical implications remain regarding the use of genome editing to develop large quantities of human HLA-matched ESCs. The previously mentioned study focuses on generating ESCs with HLA types specific to the Asian-Pacific population, which is known to have low variations in HLA types.^70^ Therefore, these biobanked ESCs are less suitable for patients with other ethnic backgrounds, and more research would be needed to develop ESCs suitable for patients of all ethnicities. Biobanks for selected ethnicities conflict with patient inclusion, which aims at ensuring equal healthcare opportunities for all. Furthermore, so far, this technique has only been tested on ESCs and there are no reported cases on HLA-matching for organoids.

### Origin of Biomaterials

Besides the ethical implications regarding the use of patient-own tissue, also the use of body foreign materials like certain biomaterials are up for debate.^76^ Thus far, the most suitable biomaterial for organoid culture is Matrigel, which is derived from a tumor cell line, and multiple mice need to be euthanized for just 10 mL. Therefore, alternatives are desirable from both an ethical and clinical perspective. One example is the use of modified poly-ethylene glycol gels that have recently been developed for the expansion and differentiation of organoids.^77-79^ Yet, there are also ethical implications that must be considered for nonanimal–derived biomaterials. Most attention has been directed to whether an immune response is elicited, but other side effects must be evaluated as well. The implanted construct will be subjected to signaling molecules, like cytokines, growth factors, ECM enzymes, and proteins, that can be different than the native environment of the transplanted tissue. Depending on the cell type, different responses can be evoked, including cell activation, proliferation, differentiation, or migration.^63^ Although often biodegradable biomaterials are implanted, which will be degraded and replaced by patient-own material, there is much uncertainty on the exact long-term risks when implanting biomaterials in the human body. Apart from biomaterials, decellularized donor livers can also be used for TE purposes. Donor livers that are deemed unsuitable for transplantation can still be used as decellularized scaffolds—provided that the quality of the ECM meets a certain standard. To not be dependent on human donor livers at all, decellularized pig livers can be used for recellularization options; however, ethical xenograft-related dilemmas may arise here.^80^

### Societal Impact

The acceptance from the public is key to developing new treatments. Often emerging technologies or fields trigger enthusiasm and expectations.^81,82^ Yet, overselling might lead to disillusionment and misconception as the technology cannot meet up to the disproportional public expectations, which can severely damage a field’s reputation.^81,82^ Therefore, Oerlemans et al^81^ propose that, while communicating final aim of CT and TE, scientists should withhold from specifying a timeframe in which these approaches will be clinically available to all. Additionally, the potential of the relatively new LRT field raises concerns for some. The objective of this field is to restore damaged liver tissue with the use of cells, biomaterials, growth factors, or a combination thereof. Yet, if we can sufficiently restore a certain function, it is highly likely that enhancing any form, function, or even lifespan of an individual can be made possible as well. Therefore, we must ensure that these technologies are merely applied to treat the disease and do not shift to improving human function beyond what is necessary to sustain or improve human health.^83^ To guarantee this (governmental), regulatory boundaries must be set but discussing those falls beyond the scope of this overview.

## ECONOMIC CONSIDERATIONS FOR ORGANOID TECHNOLOGIES

Next to assessing the clinical potential and ethical implications, timely inclusion of economic capabilities or support should be sought to increase the chance of success of bringing new treatment options to the market. Here, we compare the expected costs for CT and TE with the current standard of DLT using existing literature and calculations to provide a first insight into what factors should be considered when assessing feasibility. This cost comparison is a preliminary assessment of the proposed alternatives, and costs are based on in-house calculations and estimates. Note that costs may vary between countries, facilities, and methods of culturing.

### Preliminary Cost Assessment

**FIGURE 4.**
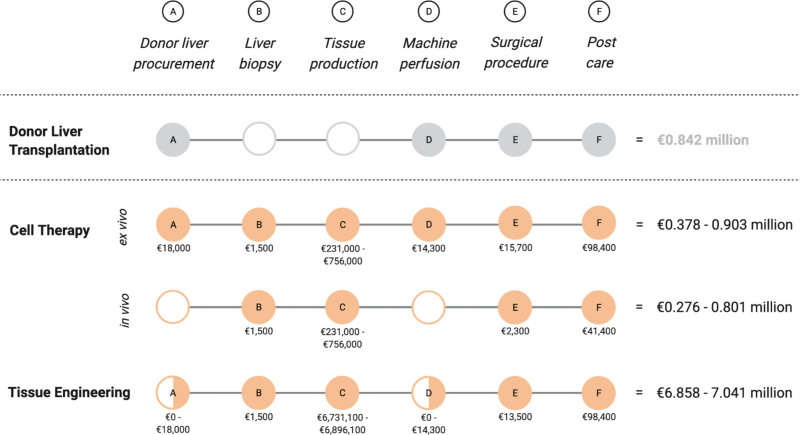
Schematic overview of the total treatment expenses per patient for organoid-based cell therapy and tissue engineering compared with the total costs of donor liver transplantation per patient. Created with BioRender.com.

DLT is a complex treatment that requires extensive professional expertise and lifelong postcare, making it one of the costliest medical procedures worldwide.^74^ Yet, the treatment is effective as it increases life expectancy by >10 y for most patients, as well as the QoL.^84,85^ Because of this, DLT is still the standard of care for ESLD patients. In 2020, the total cost for a single DLT procedure in the United States was estimated at €0.842 million ($878 400, exchange on May 12, 2022). This number includes pretransplant care (30 d), follow-up care (180 d), and drugs like immunosuppressants.^86^ Here, CT and TE will be compared with DLT, which included the following aspects (see Figure 4):

Donor liver procurement,
Liver biopsy,
Tissue production,
Machine perfusion,
Surgical procedure,
Postcare.

For all treatment options the costs for the surgical procedure (E) and postcare (F) should be considered. Yet, donor liver procurement (A) is only required in the case of DLT, CT for ex vivo repair of donor livers, and TE using the de/recellularization approach. To add, consecutive machine perfusion (D) is only required in forementioned cases because using a complete liver requires sufficient vascular perfusion, whereas for most organoids and smaller tissues, the vascular system is absent and oxygen and nutrient transport is regulated through diffusion. Furthermore, a liver biopsy (B) and tissue production (C) are required for both CT and TE. A complete breakdown of the costs can be found in **Tables S1 to S4** (**SDC**, http://links.lww.com/TP/C684) of the **Supplemental Materials** (**SDC**, http://links.lww.com/TP/C684). Organoid-based CT is expected to cost €0.276 to €0.801 million for patient treatment, and €0.378 to €0.903 million for ex vivo donor liver repair, whereas organoid-based TE is expected to cost between €6.858 and €7.041 million.^85-97^ Importantly, a lower cell number is required for CT, which significantly decreases tissue production costs. Yet, it should be noted that CT is not sufficient to treat all liver patients.

### Economies of Scale

An important factor that has not been considered is the fact that costs may change over time. Typically, as a technology develops, cost advantages occur because of incremental innovation combined with increased operational scale (ie, economies of scale). The result of this has already been witnessed for previous waves of biomedical innovation, such as for whole genome sequencing. The total costs for sequencing the first human genome were initially estimated to be around €3 billion, whereas today, this can be sequenced for around €1000 ($1000, exchange on September 22, 2022).^98,99^ It is expected that similar evolvement is applicable to CT and TE. Our initial cost estimation shows high costs related to tissue production. Yet, when these approaches gain traction, it is expected that these costs will considerably decrease because most of the tissue production costs will be reduced when operational scales increases.^89^ Yet, major bottlenecks of organoids remain quality control, reproducibility, and upscaling, as current protocols require a lot of manual labor, making it a time-consuming process susceptible to variability.^100^ To exemplify, bioreactors offer advantages over static cultures, such as preventing gradient formation (eg, of nutrients, pH, and dissolved oxygen) and cell sedimentation and increase reproducibility in a time- and cost-effective manner.^64^ Compared with static cultures, liver organoid spinner flask cultures have shown a 40-fold increase in cell numbers compared with a 6-fold increase in controls over a 2-wk period.^64^ Also, the use of automated cell expansion systems is expected to reduce labor and material costs, as culture medium usage, and spillage can be minimized.^101,102^ Additionally, costs for machine perfusion are currently substantial, but optimization might lead to cost savings as well.^90^ Thus, we expect that with advances in automation of production and perfusion and replacement of high-cost compounds, the overall expenses will significantly reduce over time and cost-effectiveness will improve.

### Organoid Value Addition

Taking a step back, it is important to address how and where organoids can be of added value in our current healthcare system. Developing new medical technologies, being pharmaceuticals, surgical interventions, or otherwise, is a costly and timely process. Alongside the development pipeline, several applications have been defined in which organoids can be used to increase (drug) development success and more efficiently allocate resources. For instance, liver organoids can be used in hepatotoxicity studies, personalized medicine approaches, and disease modeling.^103,104^ The market estimates are clearly defined for organoids used for toxicology studies, yet less for other applications. This overview solely focuses on the use of organoids for treatment purposes, of which the market estimates currently are limited. The efficacy of organoids has yet to be demonstrated in first-in-human trials before claims can be made about their value-adding properties.^105^

## OUTLOOK AND CONCLUSION

In general, triage between patients needs to be prevented. As such, the development of new alternatives is crucial to overcome the donor liver shortage. Creating alternatives through CT or TE would ensure improvement of patients’ QoL and life expectancies, given that off-the-shelf availability can be guaranteed. As for many scientific innovations, clinical, ethical, and financial implications have yet to be addressed, and an approach’s feasibility must be evaluated on the basis of the combination of all the forementioned aspects. In this overview, we have provided an outline of the clinical, ethical, and economic implications of organoid-based CT and TE for liver patients. An overview of all advantages and disadvantages per approach can be found in Table 1.

**TABLE 1.** Clinical, ethical, and economic advantages and disadvantages for organoid-based cell therapy and tissue engineering

	Clinical		Ethical		Economic	
	+	–	+	–	+	–
Organoids for cell therapy	Less invasive method if liver is partially affected	Difficult to get fully differentiated cells in vitro	Use of autologous cells	Use of allogeneic cells	Low costs for tissue production because of lower number of cells needed	
	Less time-consuming	Low engraftment efficiency in liver parenchyma		Use of Matrigel for organoid culture	Low costs related to injection of organoid cells	
	Can be completely independent of donors	Not suitable for all patients with liver diseases			Low care costs after surgery because of shorter recovery period	
	Recipient-own cells can be used					
Organoids for tissue engineering	Only alternative if all liver cell types are affected	Very time-consuming method	Use of autologous cells	Use of allogeneic cells		High costs related to engineering process
	Complex tissues, including nonparenchymal cells, can be created	Difficult to get fully differentiated cells in vitro		Use of Matrigel for organoid culture		High costs related to implanting whole liver
	Recipient-own cells can be used	No method available to fully replicate native liver		Use of porcine decellularized livers		High costs related to care after surgery

Organoids hold great promise for CT and TE as future alternative to donor organs. However, every advantage mentioned in Table 1 should be explored further, and the disadvantages must be considered before moving to clinical trials. Based on our analysis, we expect that patients having a disease that affects a single-cell function can be treated best with organoid-based CT, as it will solely restore the failing liver function. This makes the procedure less invasive, less risky, and less time-consuming. Although for patients having diseases that affect the entire liver, organoid-based TE might be the preferred solution. Yet, we believe that treating liver disorders using organoid-based CT and TE can only be achieved after optimization of engraftment and maturation of the transplanted cells.

Additionally, more attention should be directed toward developing protocols and techniques to further optimize and decrease the time required for large-scale expansion, such as the use of spinner flasks.^64^ Yet, with scaling up in vitro tissue production, the urgency for sufficient cryopreservation protocols for biobanking organoids increases as well. Furthermore, existing methods need to be refined and new methods need to be developed to be able to modify allogeneic tissue to avoid immunogenicity and replace animal-derived matrices, because many clinical and ethical implications are related to the source of cells and biomaterials. Gene editing might overcome immunogenicity problems by matching HLA types between donor and recipient. Although we acknowledge that ethical dilemmas also relate to gene editing in general, discussing these dilemmas is beyond the scope of this overview.

Above all, societal acceptance is key to developing new treatment options and is not only associated with ethical considerations but also related to economic aspects. Economic considerations are often not discussed as part of scientific articles because of the difficulty of forecasting related expenses, the impact on the local/global economy, and the societal adaptation rate of new innovations. In the economic consideration section of this overview, we have attempted to offer a cost estimation per approach. Important to note is that these numbers come with many assumptions, such as materials used to generate organoids, time spend on surgical procedures, staff required for such procedures, and associated salary costs, among others. Also, the cost estimates regarding tissue production might differ between protocols and institutions, as different materials and methods can be used to generate organoids. Here, the cost estimations are based on our own data for liver organoid cultures in stirred suspension spinner flasks. Moreover, current standard operating protocols are not yet Good Manufacturing Practice compliant or Food and Drug Administration approved. Therefore, it is important to involve private stakeholders, such as biotechnology or pharma companies in the optimization of organoid production methods.^106^ Typically, those stakeholders have the facilities and financial resources to translate new technologies into Food and Drug Administration/European Medicines Agency–approved clinical applications at a higher pace compared with academia alone.

Furthermore, it is crucial to consider the onset of continued incremental innovation and economies of scale when assessing a new innovation’s feasibility. Although forementioned estimations are too high for organoid-based therapies to become available to all, we expect these costs to significantly decrease over time as cost advantages occur because of increased operational scale.^89^ Additionally, to be able to commercialize organoids, it should be stressed that organoids can add different degrees of value to different parts of the healthcare system and the medical technology development pathway.^104-106^

However, the donor liver shortage itself is not the only limiting factor for being able to treat all liver patients, as many infrastructural bottlenecks continue to exist as well. Discussing those falls beyond the scope of this overview, but in the end, all remaining bottlenecks are related to economic constraints that hinder the upscaling of treating liver patients by replacing diseased with healthy liver tissue (whether achieved with organoids, constructs, or whole livers)—even if more tissue is available. In other words, we must continue to think about the last mile in the first step of treatment development.

## ACKNOWLEDGMENTS

The authors thank Anne-Floor de Kanter for her expert opinion on the ethical implications.

## Supplementary Material


