# Supplementary material for: Toward Transplantation of Liver Organoids: From Biology and Ethics to Cost-effective Therapy
Source: Transplantation. 2023 Jul 20;107(8):1706–17. doi: 10.1097/TP.0000000000004520 (PMC10358442; doi:10.1097/TP.0000000000004520)
Supplement: Supplementary file 1 [file tpa-107-1706-s001.pdf]

SUPPLEMENTARY MATERIALS

Preliminary cost assessment – Cell Therapy

For CT, the procurement of a donor liver (A) and machine perfusion (D) are only required in the case of *ex vivo* donor liver repair (*Can$14,878 and Can$19,417, exchange on May 12, 2022*).[68] Yet, the need for a liver biopsy (B) and consecutive tissue production (C) are required for both *ex vivo* donor liver repair and *in vivo* cell therapy (*Can$14,878, exchange May 12, 2022*).[69,70] *Habka et al*. have estimated the total costs of a complete iPSC-derived bioengineered liver to be around €26.57 million (*$27,715,000, exchange on May 12, 2022*).[67] However, their calculations are based on the costs of commercial cryopreserved iPSCs, but we believe costs can be reduced by expanding cells *in vitro* instead. Based on our own data, we estimate the production of 1 billion liver organoids in spinner flasks to be around €21,000 (see *table S2*). For CT purposes, we assume that replacing 3-10% of the total liver cell mass is sufficient, which equals ~11-36 billion organoid cells, resulting in a total of €226,800 to €756,000. Interestingly, there are already indications that companies can produce organoids at lower costs. The company *Bilitech* offers CT options to treat patients with liver disorders or repairing damaged donor livers using liver organoids to increase the organ quality upon transplantation. The company itself estimates the production costs to be at €37,400 per person (*$39,000, exchange on May 12, 2022*), which is considerably lower than our calculations. However, an independent center of excellence, the Cell and Gene Therapy Catapult, conducted an independent economic analysis of *Bilitech* in 2020. They concluded that *Bilitech* could reasonably charge up to €853,200 per patient – the exact price varies since it depends on the patient's liver disorder (*$890,000, exchange on May 12, 2022*). This price is significantly higher than the production costs estimated by the company itself but is driven by the high and rising costs of DLT.[70]

Additionally, the surgical procedure (E) to treat patients requires less time, 2 hours on average,[71] whereas DLT takes up to 12 hours.[72] Per surgical procedure, the average case costs are €382/hour, and the salary costs for surgeons and anesthesiologist are €562/hour and €213/hour, respectively (*Can$520/hour, Can$716/hour, and Can$290/hour, exchange on May 12, 2022*).[68] Related to the surgery time, the number of days spent in the hospital for the pre- and post-care (F) is also lower compared to DLT due to the less invasive nature of CT to treat patients Typically, patients are hospitalized for 24.8 days on average after donor liver transplantation,[73,74] whereas we hypothesize this will be around 2 days after CT. The average hospital cost is €2,500 per day (*$2,607/day, exchange May 12, 2022*). After surgery, immunosuppressive drug-use is required, given that allogeneic tissue is used, which is estimated to be €36,400 by *Milliman* (*$38,000, exchange on May 12, 2022*).[65] This number includes immunosuppressive drug-use after treatment for a total of 180 days, and is also used to calculate the costs for DLT. Yet, when CT is used to repair liver grafts the surgical procedure include both the time and costs to transplant the liver organoids into the donor liver, as well as transplanting the repaired donor liver into the patient. Therefore, the total time spend on the surgical procedure is 14 hours.[71,72] Since the treatment itself is similar to DLT, the recovery time and associated care costs are comparable as well.[73,74]

Preliminary cost assessment – Tissue Engineering

For TE, steps (A) to (F) are all required, given that the de-/re-cellularization approach is used to engineer new liver tissue. In the case of using hydrogels as a scaffold to seed organoids on, the procurement of a donor liver (A) is not required,[68] and tissue production (C) does not require the decellularization step but includes the purchase of hydrogels. Additionally, compared to CT, the costs for tissue production here are much higher since a liver construct is created by seeding organoids and a scaffold, whereas this scaffold is not included for CT and less cells

are used there. In the case of a split donor liver procedure, only the right lobe of the donor liver is transplanted, which is estimated to be 761 +/- 181 cm^3.[88] Therefore, we assume that a similar size of a bioengineered liver is required to restore the failing liver function. The total number of cells in the liver is estimated to be around 360 billion reaching a total size of 1470 cm^3.[76] The right lobe is approximately half of the total liver cell mass, therefore a total of 180 billion cells are needed. According to *Habkah et al.* 180 billion cells would be €13.82 million. Based on our own data, we estimate the production of 1 billion liver organoids in spinner flasks to be around €21,000 (see *table S2*). Assuming production prizes for other cell types are similar, the production of 180 billion cells comes down to €3,780,000. Yet, 44% of the total costs are related to the use of Matrigel, which is only required for 3D cultures like organoids. Both hepatocytes and cholangiocytes can be generated using organoid cultures, which constitute for 70% and 3-5% of the total liver cell mass, respectively.[75,76] Other cell types might be cultured without the need for Matrigel, which would significantly decrease the total costs. Therefore, we estimate that only 75% of the total liver cells mass requires Matrigel. Calculating the cells required for the right liver lobe alone, this comes down to 135 billion cells cultured with Matrigel, combined with the remaining 45 billion cells cultured without the use of Matrigel. Assuming other biomaterial costs are negligible, the total cost would be €3,361,500. Afterwards, all cells will be seeded on a scaffold which can either be a hydrogel or decellularized liver. Based on our own data, we estimate the costs for hydrogels to be around €8,800 on average (*see table S3*). Importantly, this only refers to the backbone without any modifications such as cell adhesion sites, degradation sites, etc. The process of decellularizing the right liver lobe costs around €173,800 (*see table S4*). Additionally, when a large tissue is engineered, perfusion (D) of the construct is required to ensure high cell viability upon transplantation. This can be done via normothermic machine perfusion using the OrganOx machine, for which the operational cost is estimated to be €14,300 per run (*Can$19,417,*

exchange on May 12, 2022).[68] Also, due to the size of the constructs we hypothesize that the surgical procedure (E) takes as much time as DLT.[72] Related to that, the number of days spend in the hospital for the pre- and post-care (F) will therefore also be comparable to DLT.[73,74] The use of immunosuppressive drugs might be required, if allogeneic tissue is used, and so the total cost for immunosuppressive drug-use is similar to calculation mentioned for CT.

Table S1 *– Breakdown of the total treatment expenses per patient per approach (cell therapy or tissue engineering). Breakdown is divided into six subcategories in chronological order: A) Donor liver procurement, B) Liver biopsy, C) Tissue production, D) Machine perfusion, E) Surgical procedure, and F) Post-care. All numbers are rounded to hundreds.*

		**Cell therapy**		**Tissue engineering**	
		Ex vivo	*In vivo*	*Hydrogel*	*Decellularized liver*
**A Donor liver procurement**[73]	Alberta reciprocal claim	€17,200	-	-	€17,200
	HOPE supplies	€800	-	-	€800
	Total costs	*€18,000*	-	-	*€18,000*
**B Liver biopsy**[74]	Percutaneous liver biopsy	€1,500	€1,500	€1,500	€1,500
	Total costs	*€1,500*	€1,500	*€1,500*	*€1,500*
**C Tissue production**[75]	Spinner flask 11-36 billion cells	€231,000-€756,000	€231,000-€756,000	-	
	Spinner flask 180 billion cells	-	-	€6,722,280	€6,722,280
	Decellularizing donor liver	-	-	-	€173,800
	Hydrogel purchase	-	-	€8,800	-
	Total costs	€231,000-€756,000	€231,000-€756,000	*€6,731,080*	*€6,896,080*
**D Machine perfusion**[73]	OrganOx operative costs	€14,300	-	€14,300	€14,300
	Total costs	*€14,300*	-	*€14,300*	*€14,300*

**E Surgical procedure**[76,77]	Average case costs	€382/hour * 14 hours = €5,300	€382/hour * 2 hours = €800	€382/hour * 12 hours = €4,600	€382/hour * 12 hours = €4,600
	Surgeon costs	€526/hour * 14 hours = €7,400	€526/hour * 2 hours = €1,100	€526/hour * 12 hours = €6,300	€526/hour * 12 hours = €6,300
	Anesthesia costs	€213/hour * 14 hours = €3,000	€213/hour * 2 hours = €400	€213/hour * 12 hours = €2,600	€213/hour * 12 hours = €2,600
	Total costs	*€15,700*	*€2,300*	*€13,500*	*€13,500*
**F Post-care**[68,78-80]	Average hospital costs	€2,500 * 24.8 days = €62,000	€2,500/day * 2 days = €5,000	€2,500 * 24.8 days = €62,000	€2,500 * 24.8 days = €62,000
	Immunosuppression costs	€36,400	€36,400	€36,400	€36,400
	Total costs	*€98,400*	*€41,400*	*€98,400*	*€98,400*
	**Cumulative total costs**	**€378,900-€903,900**	**€276,200-€801,200**	**€6,858,800**	**€7,041,800**

Table S2 *– Breakdown of the total costs for spinner flask cultures per 1 billion cells with and without Matrigel for both CT and TE.*

Cumulative total numbers are rounded to hundreds.

All numbers mentioned here are based on the protocol used in the publication from *Schneeberger et al.* (2019)

- Recommended seeding density in spinner flasks (Corning #4500-250) is ~2.5 million cells
- A confluent flask has a cell density of ~100 million cells, 22 mL Matrigel (BD Biosciences #356231), and 195 mL medium (StemCell #100-0385)

With Matrigel

**Number of flasks**	1 billion organoid cells	*with*	100 million cells/flask	*equals*	10 spinner flasks
	€562	*per a total of*	1 flask	*equals*	€5,620 in total
**Amount of biomaterial**	22 mL Matrigel per flask	*with*	10 spinner flasks	*equals*	220 mL Matrigel
	€420	*per a total of*	10 mL Matrigel	*equals*	€9,240 in total
**Amount of medium**	195 mL medium per flask	*with*	10 spinner flasks	*equals*	1950 mL medium
	€314	*per a total of*	100 mL medium	*equals*	€6,123 in total
		Cumulative costs for 1 billion cells			*€21,000 in total*

Without Matrigel

**Number of flasks**	1 billion cells	*with*	100 million cells/flask	*equals*	10 spinner flasks
	€562	*per a total of*	1 flask	*equals*	€5,620 in total
	195 mL medium per flask	*with*	10 spinner flasks	*equals*	1950 mL medium

**Amount of medium**	€314	*per a total of*	100 mL medium	*equals*	€6,123 in total
		Cumulative costs for 1 billion cells			*€11,700 in total*

Cell Therapy

**Cells cultured with Matrigel**	11-36 billion cells		
	€21,000 per a total of 1 billion cells	*equals*	€231,000-756,000
	**Cumulative total costs**		**€231,000-756,000 in total**

Tissue Engineering

**Cells cultured with Matrigel**	(70% hepatocytes + 3-5% cholangiocytes) / 2	*equals*	135 billion cells
	€21,000 per a total of 1 billion cells	*equals*	€2,835,000
**Cells cultured without Matrigel**	360 / 2 - 135 billion cells	*equals*	45 billion cells
	€11,700 per a total of 1 billion cells	*equals*	€526,500
	**Cumulative total costs**		**€3,361,500 in total**

Table S3 *– Breakdown of the total costs for hydrogel-based liver tissue.*

- The following calculations are true for a construct size of 10 cm^3
- We assume 500 µL hydrogel is needed per cm^3 construct (5 mL for 10 cm^3)
- Four different hydrogels are used to calculate an average
  - GEL-MA (Sigma-Aldrich #900496)
  - PEG (Sigma-Aldrich #P7181)
  - Alginate (Sigma-Aldrich #901953)

**GEL-MA**	5 mL GEL-MA needed				
	€232	*per a total of*	10 mL GEL-MA	*equals*	€116 in total
**PEG**	5 mL PEG needed				
	€135	*per a total of*	25 mL PEG	*equals*	€67.50 in total
**Alginate**	5 mL alginate needed				
	€329	*per a total of*	10 mL Alginate	*equals*	€164.50 in total

Average cumulative costs for 10 cm^3	*€116 in total*
**Average cumulative total costs for 761 cm^3**	**€8,800 in total**

Table S4 *– Breakdown of the total costs for decellularizing complete liver tissue. Cumulative total numbers are rounded to hundreds.*

All numbers mentioned here are based on the protocol used in the publication from *Mazza et al.* (2015)

- The following calculations are true for a construct size of 10 cm3, with a perfusion rate of 1 mL/min
- Different perfusion steps with either PBS, SDS or Triton X100 need to be performed
- The total perfusion time per compound:
  - PBS (Sigma-Aldrich #P5493) = 370 min (with a starting volume of 8 liters PBS)
  - SDS (Sigma-Aldrich #71736) = 3290 min (at different concentrations)
  - Triton X100 (Sigma-Aldrich #X100) = 30 min

**PBS**	8370 mL needed				
	€635	*per a total of*	4000 mL	*equals*	€1,329 in total
**SDS**	3290 mL needed				
	€143	*per a total of*	500 mL	*equals*	€941 in total
**Triton X100**	30 mL needed				
	€49,40	*per a total of*	100 mL	*equals*	€15 in total
		Cumulative costs for 10 cm^3			*€2,284 in total*
		**Cumulative total costs for 761 cm^3**			**€173,800 in total**
